# BUDDY-system: A web site for constructing a dataset of protein pairs between ligand-bound and unbound states

**DOI:** 10.1186/1756-0500-4-143

**Published:** 2011-05-22

**Authors:** Mizuki Morita, Tohru Terada, Shugo Nakamura, Kentaro Shimizu

**Affiliations:** 1 Department of Fundamental Research, National Institute of Biomedical Innovation (NIBIO), 7-6-8 Saito Asagi, Ibaraki, Osaka 567-0085, Japan; 2 Institute for Bioinformatics Research and Development (BIRD), Japan Science and Technology Agency (JST), 4-1-8 Honcho, Kawaguchi, Saitama 332-0012, Japan; 3 Agricultural Bioinformatics Research Unit, The University of Tokyo, 1-1-1 Yayoi, Bunkyo, Tokyo 113-8657, Japan; 4 Department of Biotechnology, The University of Tokyo, 1-1-1 Yayoi, Bunkyo, Tokyo 113-8657, Japan

## Abstract

**Background:**

Elucidating molecular recognition by proteins, such as in enzyme-substrate and receptor-ligand interactions, is a key to understanding biological phenomena. To delineate these protein interactions, it is important to perform structural bioinformatics studies relevant to molecular recognition. Such studies require a dataset of protein structure pairs between ligand-bound and unbound states. In many studies, the same well-designed and high-quality dataset has been used repeatedly, which has spurred the development of subsequent relevant research. Using previously constructed datasets, researchers are able to fairly compare obtained results with those of other studies; in addition, much effort and time is saved. Therefore, it is important to construct a refined dataset that will appeal to many researchers. However, constructing such datasets is not a trivial task.

**Findings:**

We have developed the BUDDY-system, a web site designed to support the building of a dataset comprising pairs of protein structures between ligand-bound and unbound states, which are widely used in various areas associated with molecular recognition. In addition to constructing a dataset, the BUDDY-system also allows the user to search for ligand-bound protein structures by its unbound state or by its ligand; and to search for ligands by a particular receptor protein.

**Conclusions:**

The BUDDY-system receives input from the user as a single entry or a dataset consisting of a list of ligand-bound state protein structures, unbound state protein structures, or ligands and returns to the user a list of protein structure pairs between the ligand-bound and the corresponding unbound states. This web site is designed for researchers who are involved not only in structural bioinformatics but also in experimental studies. The BUDDY-system is freely available on the web.

## Findings

**Figure 1:**
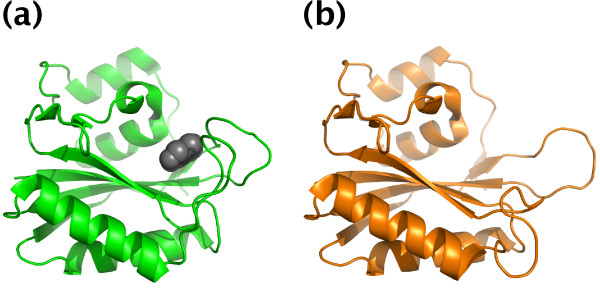
**Example of a protein structure pair between ligand-bound and unbound states**. (a) A ligand-bound state structure of 6-hydroxymethyl-7,8-dihydropterin pyrophosphokinase (HPPK) ([PDB:1HQ2]) and (b) an unbound state structure ([PDB:1IM6]). The ligand is represented by dark spheres. The BUDDY-system allows users to search for this type of pair by its ligand as a search query, bound states and ligands by its unbound state, and unbound states from its bound state. The user can input 1 query or a set of such queries as a dataset.

Elucidating molecular recognition by proteins is one of the keys to understanding biological phenomena. Structural bioinformatics studies relevant to molecular recognition, such as analysis of conformational changes upon ligand binding [1-4], development of methods for predicting ligand binding sites [5-7], and development of molecular docking tools [8-10], require a dataset of protein structure pairs between ligand-bound and unbound states (Figure 1).

**Figure 2:**
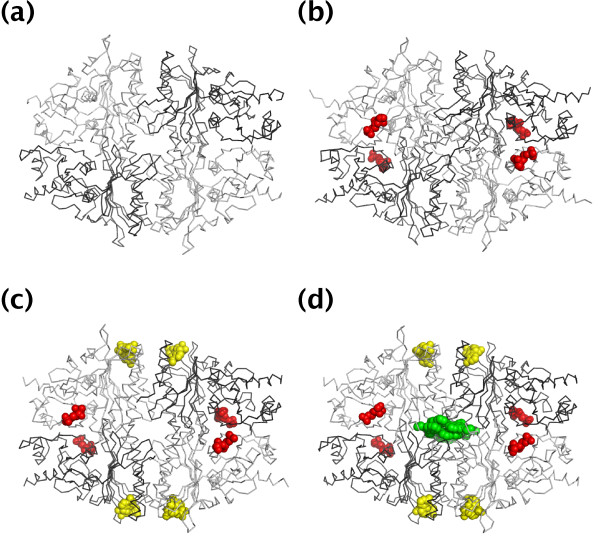
**Examples illustrating the difficulty in defining a ligand**. F16BPase tetramer (a) in free form ([PDB:2FBP]), (b) with F6P (red sphere) in the active site ([PDB:1RDX]), (c) with F6P and AMP (yellow sphere) in the allosteric site ([PDB:1FBP]), and (d) with F6P, AMP, and PFE (green sphere) in the non-native allosteric site ([PDB:1KZ8]). Although [PDB:1RDX] is in a ligand-bound state against [PDB:2FBP], it is also in a ligand-unbound states against [PDB:1FBP]. Further, although [PDB:1FBP] is in a ligand-bound state against [PDB:2FBP] and [PDB:1RDX], it is also in a ligand-unbound state against [PDB:1KZ8]. If a ligand is defined specifically as "HETATM molecules except for low-molecular ions," all entries but [PDB:2FBP] are obtained in a ligand-bound state. The flexible ligand definition in the BUDDY-system enables the user to obtain all possible ligand-bound and unbound states pairs of F16BPase.

The BUDDY-system features a flexible definition of a ligand and allows the user to change various options via its web interface. The BUDDY-system is based on a premise that differs from existing structural bioinformatics systems in terms of what is considered a ligand. In previous studies, a ligand was defined as all heterogeneous (HETATM) molecules in the Protein Data Bank (PDB) [11] files [1,12], all HETATM molecules except for low-molecular ions (e.g., Zn^2+^, Mn^2+^, PO_4_^3-^, and SO_4_^2-^) [4], or HETATM clusters forming many inter-atomic contacts with protein atoms [13]. This variety of ligand definitions implies that it is very difficult to specifically define a ligand. Here, we define a ligand as molecules that can dissociate from proteins; consequently, a certain protein can be found with a ligand in some entries in PDB and without it in other entries. Under this definition, a ligand is not determined specifically but instead depends on each pair of PDB entries. For example, the structure of fructose-1,6-bisphosphatase (F16BPase) [14], which catalyzes the hydrolysis of d-fructose 1,6-bisphosphate (FBP) to d-fructose 6-phosphate (F6P) and phosphate (Pi), has been demonstrated several times in different binding states (Figure 2): F16BPase in free form ([PDB:2FBP]); with F6P in the active site ([PDB:1RDX]); with F6P and adenosine monophosphate (AMP) in the allosteric site ([PDB:1FBP]); and with F6P, AMP, and the anilinoquinazoline inhibitor (PFE) in the non-native allosteric site ([PDB:1KZ8]). If a ligand is defined specifically as "HETATM molecules except for low-molecular ions," [PDB:2FBP] would be reported as existing in the ligand-unbound state and all the others in the ligand-bound state. However, although [PDB:1RDX] exists in the ligand-bound state against [PDB:2FBP], it also exists in the ligand-unbound state against [PDB:1FBP] and [PDB:1KZ8]. Likewise, while [PDB:1FBP] is in the ligand-bound state against [PDB:2FBP] and [PDB:1RDX], it is also present in a ligand-unbound state against [PDB:1KZ8]. The flexible ligand definition in the BUDDY-system enables the user to obtain all possible ligand-bound and unbound state pairs of F16BPase.

We plan to implement more advanced search options in the future, such as protein sequence similarity search and chemical structure search from SMILES.

## Methods

**Figure 3:**
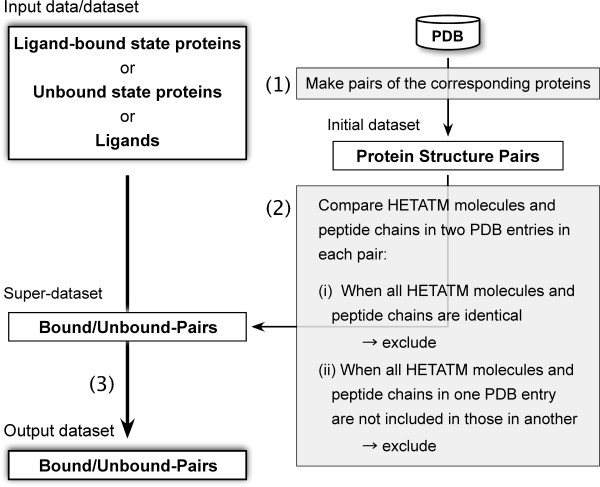
**Schematic diagram illustrating the construction of a dataset in the BUDDY-system**. The process of constructing a pair dataset consists of the following 3 steps: (1) all pairs of the same proteins or homologues are obtained from the entire PDB entries to prepare an initial dataset, (2) protein structure pairs of ligand-bound and unbound states are screened from the initial dataset to prepare a super dataset, and (3) the pairs that fit the user's request are selected from the super dataset after the user submits a request.

The procedure for constructing a dataset of protein pairs between ligand-bound and unbound states (called bound/unbound-pairs) in the BUDDY-system consists of the following 3 steps: (1) finding all pairs of the same proteins or homologues in all the PDB entries to prepare an initial dataset, (2) screening bound/unbound-pairs from the initial dataset to prepare a super dataset, and (3) finding suitable pairs for the user's request from the super dataset after the user submits a request (Figure 3). The first 2 steps are carried out in advance, and the third step can be achieved after the user enters input data. The details are as follows. (1) The BUDDY-system finds pairs of the same proteins or homologues from all of the PDB entries based on their sequence identity to prepare an initial dataset (the sequence identity threshold can be specified by the user via the web interface). Here, a chain shorter than *N *amino acids is defined as "a peptide chain" and is considered a ligand (*N *can be specified by the user via the web interface). This option is useful, especially when a protein has short amino acid chains that are essential for its function (e.g., insulin). (2) Next, the BUDDY-system screens the bound/unbound-pairs to prepare a super dataset from the initial dataset. Initially, the BUDDY-system compiles HETATM lists of both PDB entries in a pair, respectively. Here, when an HETATM molecule appears more than once in a PDB entry, it is listed only once in its HETATM list. Furthermore, HETATM molecules that are defined as "not considered as a ligand" will be excluded from the HETATM list. If the PDB file has chains shorter than *N *amino acids in the ATOM record (*N *can be decided by the user via the web interface), they are considered "peptide chains." The BUDDY-system then compares 2 HETATM lists and peptide chains from 2 PDB entries in a pair and judges whether this pair is a bound/unbound-pair in the following manner: (2-i) when the contents of 2 HETATM lists and peptide chains are identical, this pair is not regarded as a bound/unbound-pair; and (2-ii) when those are not identical, a pair is a bound/unbound-pair if 1 HETATM list is included in another list. (3) Finally, after the user inputs ligand-bound state protein structures, unbound state protein structures, or ligands into the BUDDY-system, bound/unbound-pairs that fit the user's request are selected from the super dataset and are returned to the user. The user can (3-i) upload their own datasets including a PDB ID list of ligand-bound state protein structures, unbound state protein structures, or a HETATM ID list of ligands; (3-ii) choose one of the readymade datasets of ligand-bound state protein structures, such as BindingDB [15] and PLD [16] (whose use of each has been generously permitted by the authors listed in references 14 and 15, respectively); or (3-iii) input one PDB ID of a ligand-bound state protein structure or an unbound state protein structure, or one HETATM ID of a ligand. The file formats of the input and output datasets are described on the BUDDY-system website. The parameters that the user can select are the cut-off value of X-ray resolution, the sequence identity when making a bound/unbound-pair, and the definition of a peptide chain.

## Example Usage

Here, we show examples of using the BUDDY-system. Table 1 shows the results obtained from the BUDDY-system when a list of PDB entries of ligand-bound state proteins, which were obtained from various databases or datasets available on the Internet, were input with the following default parameters: X-ray resolutions equal to or better than 2.5 Å were allowed, a sequence identity between ligand-bound and unbound state protein equal to 100% was used, and chains shorter than 30 amino acids were considered peptide ligands. In the example shown in Table 1, when PDB entries obtained from BindingDB were input, at least 1 corresponding unbound state entry was found for 484 of 1,485 input ligand-bound state protein entries, and the number of total pairs was 4,629. Interestingly, at least 1 unbound state PDB entry was found for approximately 30% of the input ligand-bound state protein structures for any of the datasets in Table 1. Additionally, a large portion of these ligand-bound state structures was paired with only 1 corresponding unbound state protein structure. Although this number of returned pairs would increase or decrease depending on the parameters used, these examples in Table 1 demonstrate that a dataset of bound/unbound-pairs can be readily obtained with the BUDDY-system. The datasets obtained here are essential for elucidating molecular recognition by proteins in studies that investigate conformational changes involved in enzyme reactions, developments of ligand binding site prediction, and components involved in molecular docking. The BUDDY-system is the first web site that the authors are aware of that supports the construction of such a dataset according to the user's input dataset and parameters. In addition, because the ligand is allowed a more flexible definition, this web server is useful to exhaustively search for ligands or ligand-bound and unbound state structures that are of interest to the user.

**Table 1:** Summary of the results obtained using the BUDDY-system against various datasets

Input dataset^a^	Version^b^	The number of protein structures		
		
		Input^c^	Output A^d^	Output B^e^
AffinDB [17]	2008-03-17	476	157	2,257
BindingDB [15]	2008-03-17	1,485	484	4,629
PDBbind [18]	v2007	3,124	996	7,000
PLD [16]	v1.3	485	137	1,277
CCDC/Astex Test Set [8]	-	305	91	611
Astex Diverse Set [10]	-	85	25	170
Non-redundant^f^	-	4,544	1,172	7,901

This table shows the results obtained from the BUDDY-system when various databases were input as lists of ligand-bound states proteins. Taking BindingDB as an example, when 1,485 PDB entries obtained from BindingDB were input as a ligand-bound state, at least 1 corresponding unbound state PDB entry was found for 484 of 1,485 input entries; a total of 4,629 pairs were obtained.

^a^Each input dataset contains a list of PDB entries of ligand-bound state proteins.

^b^The version of the input dataset or the date when the input dataset was downloaded.

^c^The number of ligand-bound state protein structures in each input dataset.

^d^The number of unique proteins that were paired with at least one ligand-unbound state entry by the BUDDY-system.

^e^The number of total pairs.

^f^Union of all ligand-bound state protein datasets.

## Availability and Requirements

The BUDDY-system is freely available at URL http://www.bi.a.u-tokyo.ac.jp/services/buddy/.

## Abbreviations

AMP: Adenosine monophosphate; F16BPase: Fructose-1,6-bisphosphatase; F6P: d-fructose 6-phosphate; HPPK: 6-Hydroxymethyl-7,8-dihydropterin pyrophosphokinase; PDB: Protein Data Bank; Pi: Phosphate.

## Competing interests

The authors declare that they have no competing interests.

## Authors' contributions

MM developed the concept and designed the algorithm and its implementation. TT provided valuable suggestions on the manuscript. SN contributed to the implementation of the web site. KS reviewed and tested the software. All authors read and approved the final version of the manuscript.
